# Cohort Profile: The Birhan Health and Demographic Surveillance System

**DOI:** 10.1093/ije/dyab225

**Published:** 2021-11-09

**Authors:** Delayehu Bekele, Bezawit Mesfin Hunegnaw, Chalachew Bekele, Kimiko Van Wickle, Fisseha Tadesse, Frederick G B Goddard, Yahya Mohammed, Sarah Unninayar, Grace J Chan

**Affiliations:** Department of Obstetrics and Gynecology, St. Paul’s Hospital Millennium Medical College, Addis Ababa, Ethiopia; Department of Pediatrics and Child Health, St. Paul’s Hospital Millennium Medical College, Addis Ababa, Ethiopia; Birhan HDSS, St. Paul’s Hospital Millennium Medical College, Addis Ababa, Ethiopia; Department of Epidemiology, Harvard T.H. Chan School of Public Health, Boston, MA, USA; Department of Obstetrics and Gynecology, Debire Birhan Referral Hospital, Debire Birhan, Ethiopia; Department of Epidemiology, Harvard T.H. Chan School of Public Health, Boston, MA, USA; Birhan HDSS, St. Paul’s Hospital Millennium Medical College, Addis Ababa, Ethiopia; Department of Epidemiology, Harvard T.H. Chan School of Public Health, Boston, MA, USA; Department of Pediatrics and Child Health, St. Paul’s Hospital Millennium Medical College, Addis Ababa, Ethiopia; Department of Epidemiology, Harvard T.H. Chan School of Public Health, Boston, MA, USA; Division of Medical Critical Care, Boston Children’s Hospital, Department of Pediatrics, Harvard Medical School, Boston, MA, USA

Key Features

- The Birhan Health and Demographic Surveillance System (HDSS) was established in 2018 to set up a platform for community- and facility-based research and research training. The specific objectives at inception are to generate high-quality data on the health and demographic profile of a rural area of Ethiopia, with a specific focus on maternal and child health.
- The site is located in the North Shewa Zone of the Amhara Region, 130 km north of the capital Addis Ababa, and has 18 933 households and a population of 77 766 at the baseline survey. Over 82.6% of the population is rural, residing in a wide range of topographic and climatic environments.
- All houses are geocoded; data are collected every 3 months on: sociodemographic status; housing construction material; economics and asset ownership; care-seeking behaviours; immunization status; water and sanitation access; and key demographic events such as births, deaths, marital status changes, in-migration and out-migration.
- Ongoing studies include pregnancy surveillance, estimating causes of mortality using verbal autopsy, determining immunization coverage and evaluating access to water and sanitation infrastructure.

## Why was the Health and Demographic Surveillance System (HDSS) set up?

Although there has been significant progress in reducing maternal and child mortality over the past few decades, the rates remain high globally. Nearly 94% of maternal mortality occurs in low-middle income country (LMIC) settings and arises from largely preventable causes.^1^ In Ethiopia, the maternal mortality rate is 412/100 000 live births, amounting to 14 000 maternal deaths each year.^2^^,^^3^ Sub-Saharan Africa is the region with the highest under-five mortality, and Ethiopia is one of five countries which contributed to more than half of the global under-five deaths in 2018.^4^ Ethiopia is also one of 10 countries accounting for more than half of global neonatal deaths with 80 000 deaths each year.^5^ Complete, timely and accurate data are essential to understand the causes and underlying risk factors of maternal and child morbidity and mortality. These data and the resulting knowledge generated are subsequently key to the development of effective interventions to improve health outcomes. Currently, health data in Ethiopia remain limited in scope and quality, and data collected at community and health facility levels are not linked.

The Birhan HDSS (Birhan meaning ‘light’ in Amharic), was established as a sentinel field site to generate evidence on maternal and child health in Ethiopia. The Birhan HDSS collects socioeconomic, demographic and health data in the defined catchment population to improve morbidity and mortality surveillance. Nested within the Birhan HDSS is a pregnancy and birth cohort. Together, the Birhan HDSS and cohort provide detailed data on time trends in maternal and child health, to better understand the aetiologies of disease and predict risk. The platform was designed to support nested studies to assess the effects of exposures and interventions on outcomes. Information on vital events such as births and deaths builds the foundation for equitable health policies.

The study has received the following ethical approvals from: St. Paul’s Hospital Millennium Medical College (PM23/274), Boston Children’s Hospital (P00028224) and Harvard School of Public Health (19–0991).

## Where is the HDSS area?

**Figure 1:**
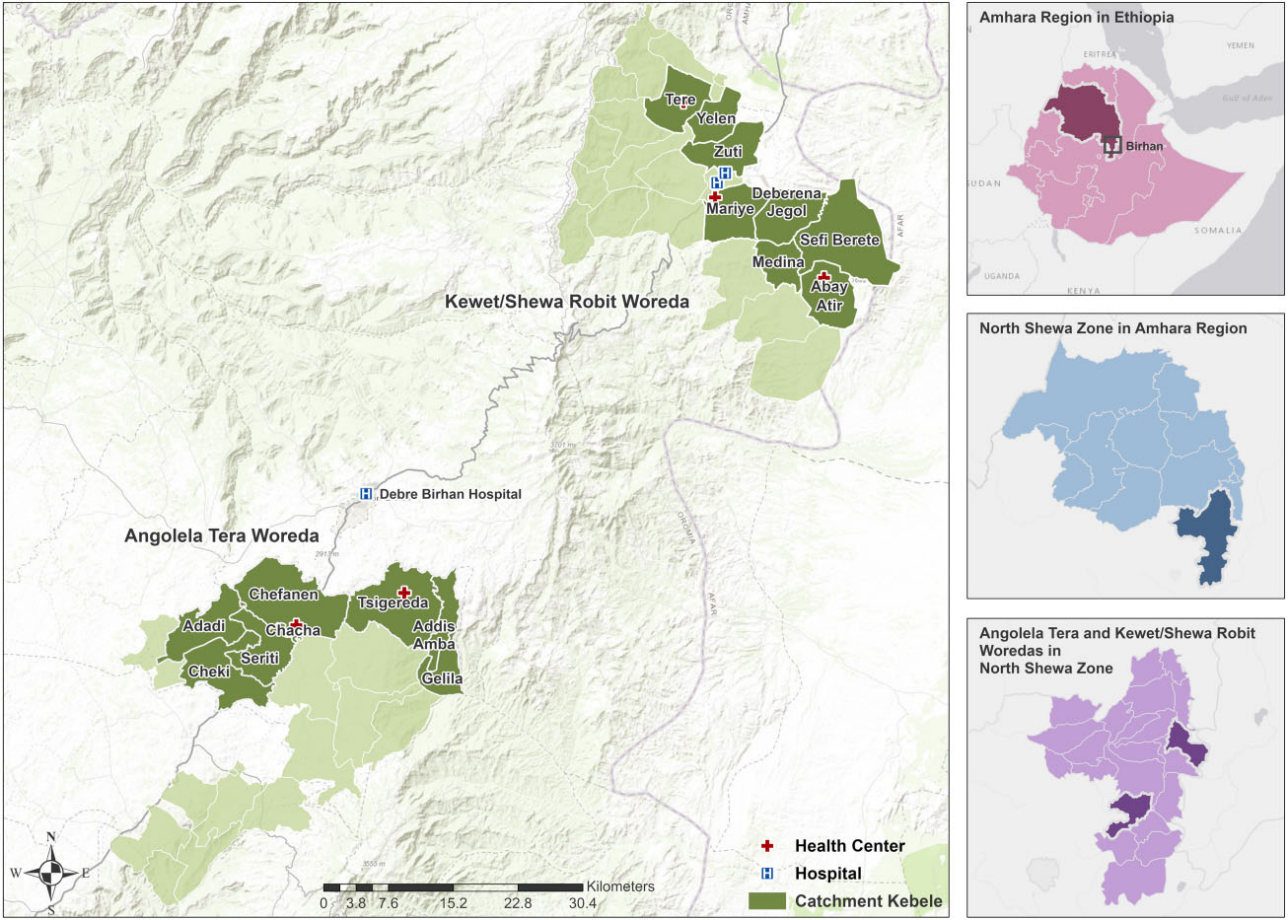
Map of the Birhan Health and Demographic Surveillance System

The Birhan HDSS was established in Ethiopia, a landlocked country in East Africa bordering Djibouti, Eritrea, Kenya, Somalia, South Sudan, and Sudan. The HDSS is located in North Shewa Zone, one of the 10 zones in Amhara Region (Figure 1). The zone is bordered on the south and west by Oromia Region, on the north and northeast by South Wollo Zone and Oromia Zone of Amhara Region, and on the east by Afar Region.^6^ The capital city of the zone, Debre Birhan, is located 130 km north of Addis Ababa, the country’s capital.

To select the site, the study team conducted rapid assessments of surrounding areas within 300 km of Addis Ababa, to evaluate these based on a set of selection criteria including the distribution of diseases, representative mix of rural and urban kebeles, highland and lowland topography, diversity of ethnicities and safety. We also considered recommendations from the zonal health bureaus.

The study site consists of two districts—Angolela Tera and Kewet/Shewa Robit—covering a total area of 636 km^2^. Each district has eight kebeles (the lowest administration unit). Of the 16 total kebeles, two are urban and the remaining 14 are rural. The study kebeles in Angolela Tera District are located 111 km north of Addis Ababa and are predominantly highland, with an altitude ranging from 1600 m to 3030 m above sea level.^7^ The monthly minimum average temperature is -0.1°C, maximum average temperature is 22.9°C and the monthly average rainfall is 25 mm (range 0–120 mm).^8^ The Kewet/Shewa Robit study kebeles are located 220 km north of Addis Ababa and are predominantly at lower elevation, with an altitude ranging from 1093 m to 2155 m above sea level.^7^ The monthly average temperature ranges from a minimum of 10.2°C to a maximum of 36.4°C, and the monthly average rainfall is 23 mm (range 0–80 mm).^8^

Most residents are farmers, with rain-based farming in the Angolela Tera kebeles and irrigation-based and semi-pastoralist types of practices in the Kewet/Shewa Robit kebeles. There are 47 primary and three secondary schools, 53 churches and 29 mosques in the study area. The HDSS site includes 16 health posts, five health centres, one primary hospital and one private hospital. Debre Birhan Hospital serves as a referral site for all facilities in the study catchment.

Following an open prospective cohort design, the Birhan HDSS routinely collects information on health and demographic events including migration (i.e. in-migration, out-migration and internal moves), births and deaths, marital status, household wealth, types of employment, water supply and sanitation access and child health. The Birhan HDSS conducts pregnancy surveillance among women aged 15–49 years. Pregnant women and children aged under 2 years are enrolled into a pregnancy and birth cohort with more frequent follow-ups (both at home and at health facilities) to assess exposures, outcomes, disease symptoms and anthropometrics.

## Who is covered by the HDSS and how often have they been followed up?

The Birhan HDSS covers all eligible residents in the defined catchment area. To identify and enumerate eligible residents, a baseline household survey was conducted from May to August 2018. The baseline survey identified all houses—defined as a structure made from permanent building material. Houses were enumerated using a unique 10-character identification system. After obtaining consent, a numbering plate was affixed to each door. A household was defined using the definition as members who ‘share a common pot’ for cooking.^9^ Members of the household were defined as individuals having a dwelling within the household and having lived in the household for at least the 3 months preceeding the survey. This included students with temporary residence elsewhere but returning home for weekends or for vacation.

Each household and household member has a unique identification number to allow for longitudinal follow-up. New individuals and households are added to the study through birth or in-migration into the study area. The study population is visited every 3 months at home and the health and demographic data are updated by trained study data collectors.

## What has been measured and how have the HDSS databases been constructed?

During the baseline survey, data collectors went door to door in each kebele to collect data on household location (including GPS coordinates), sociodemographic status, housing construction material, economics and asset ownership, care-seeking behaviours, water and sanitation access and practices, previous pregnancies, and birth histories for the past year (Table 1). During each subsequent round of house-to-house data collection every 3 months, data on key demographic events such as births, deaths, marital status changes, in-migration and out-migration are collected for all enumerated household members. Weight, height and mid upper arm circumference (MUAC) measures are collected for women of reproductive age (15–49). Data collectors conduct pregnancy surveillance among married women of childbearing age by asking pregnancy screening questions. Urine pregnancy tests are done for women who screened positive.^10^ To assess the prevalence of child morbidities during routine surveillance, data collectors ask mothers/caregivers to recall clinical symptoms of diarrhoea, pneumonia and febrile illnesses occurring within the past 2 weeks.^11–15^

**Table 1:** Data collection activities at baseline and follow-up rounds of the Birhan Health and Demographic Surveillance System

Area of study	Description
1. Baseline household census	
Location	House address, GPS^a^ coordinates, household head
Household	Household size, members of household (women of reproductive age, children)
Individual	Age, sex, ethnicity, marital status, education, occupation
Housing	House ownership, dimensions, construction and characteristics, number of rooms and purpose, cooking practices, power supply, distance from health facilities
Economics	Household income and assets, household expenditure
WASH	Water (source, access), toilet facilities (type, access, ownership), hand-washing practices, waste disposal
2. Health and demographic surveillance follow-up rounds	
Birth	Date and place of birth, sex, birth outcome
Death	Date and place of death, health care received, perceived cause of death
In-migration	Date of in-migration, place of previous residence, reason for in-migration, individual information for each in-migrant
Out-migration	Date of out-migration, destination, reason for out-migration
Marital status update	Change in marital status
Women of reproductive age	Anthropometry (weight, height, MUAC^c^), diet, smoking and alcohol intake, history of pregnancy and pregnancy outcomes, contraceptive use
Pregnancy screen	Pregnancy screen questions, urine pregnancy test
Child health and morbidity	Vaccination history (date and name of vaccine), availability of vaccination card, period prevalence of childhood illnesses, treatment provided
3. Verbal autopsy	
WHO^d^ Verbal Autopsy Questionnaire for age categories (0-28 days, 4 weeks to 12 years, >12years)	Date and place of death, details of symptoms and signs, care received, history of injury, cause of death

a GPS: Global Positioning System.

b WASH: Water, Sanitation and Hygiene

c MUAC: Mid-upper arm circumference.

d WHO: World Health Organization.

To better understand causes of mortality, verbal autopsy (VA) data are collected using tools adopted from standard World Health Organization (WHO) VA questionnaires for stillbirths, neonates, infants, children and adults.^16^ A separate dedicated team of verbal autopsy data collectors conduct VA interviews by visiting caregivers of the deceased, after a mourning period of 6 weeks.

The Birhan HDSS uses an electronic data collection system, developed as a customized version of Open Data Kit (ODK), for longitudinal and relational data collection. The system synchronizes data between surveillance data collectors and other nested studies.^17^ The database is constructed in a three-level hierarchical tier (i.e. individual, household, village). Data quality checks and logic are built into the data entry tools and data system. At the end of each round, error reports are discussed and data issues resolved before starting the next survey round.

The Birhan HDSS platform also includes a pregnancy and birth cohort to investigate time-varying exposures and associations with maternal and child health outcomes. A dedicated team of maternal and child health data collectors, based at study health facilities and in the community, enrolls pregnant women identified through pregnancy surveillance into an open pregnancy and birth cohort. During pregnancy and the postpartum period, community and health facility maternal and child health data collectors follow women at home and at facilities, based on a schedule detailed in an additional protocol for the cohort study. Pregnancy surveillance includes ultrasound gestational age dating and measurement of adverse pregnancy outcomes, which will be used to develop pregnancy risk stratification models.

## What has it found? Key findings and publications

**Figure 2:**
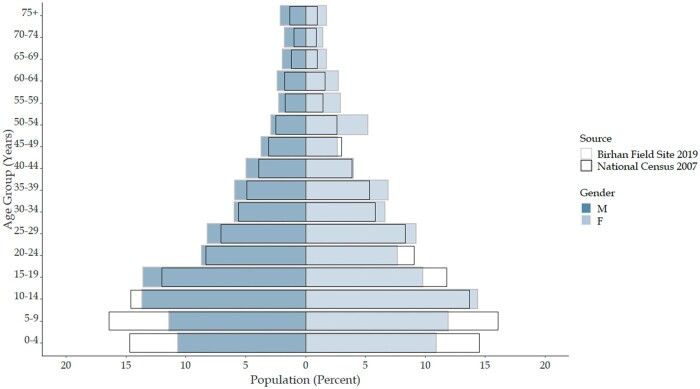
Population pyramid of Birhan Health and Demographic Surveillance System by age and sex, January-December 2019, superimposed on the population pyramid from 2007 national census

During the Birhan HDSS baseline survey in May 2018, a total of 79 653 individuals and 19 957 households were identified. Quarterly follow-up rounds were conducted in January, April, July and October of 2019. The 2019 mid-year population was 77 766 individuals living in 18 933 households (Table 2). The rural population contributes to 82.6% of the total. The average number of individuals per household was 4.1. The population is young with a broad-based population pyramid (Figure 2). The population pyramid is narrower at the base compared with the latest national census conducted in 2007. The change can be explained by the declining total fertility rate which has decreased from 5.9 children per woman in 2005 to 4.6 children per woman in 2016.^2^^,^^18^^,^^19^

**Table 2:** Demographic characteristics of Birhan Health and Demographic Surveillance System, 2019

Characteristics	Number
Mid-year population	77 766
Number of households	18 933
Rural population	82.6%
Average household size	4.1
Sex ratio at birth (male to female)	1.03
Sex ratio (male to female)	1.08
Median age (years)	21.1
Percent aged under 5 years	11.0%
Percent aged under 15 years	36.0%
Dependency ratio	0.72
Young dependency ratio	0.62
Old dependency ratio	0.10
Percent women of reproductive age (15–49 years)	22.0%

The median age is 21 years [interquartile range (IQR) 11.0–36.9]. Children under 15 years of age account for 36.0% of the population. The male to female ratio is 1.07, with females accounting for 48.2% of the population. This corresponds well with the projected national ratio of 1.09.^20^

The site has a total fertility rate of 4.8 births per woman (Table 3), which is slightly higher than the national rate of 4.6 births per woman and significantly higher than the Amhara Region-specific rate of 3.7 births per woman.^2^ This may reflect the rural majority in the study population.

**Table 3:** Vital rates among study population at the Birhan Health and Demographic Surveillance System, 2019

Characteristics	Rate
Early neonatal mortality rate per 1000 live births	17.2
Neonatal mortality rate per 1000 live births	27.6
Infant mortality rate per 1000 live births	42.1
Under-five mortality rate per 1000 live births	46.8
Crude birth rate per 1000 people	24.7
Crude death rate per 1000 people	5.4
Total fertility rate per woman	5.0

The under-five mortality rate of 47 deaths per 1000 live births (Table 3) is lower than the national rate of 55 deaths per 1000 live births reported in the 2019 Ethiopia Mini Demographic and Health Survey (EMDHS).^21^ The neonatal mortality rate of 28 deaths per 1000 live births and infant mortality rate of 42 deaths per 1000 live births are comparable to the 30 and 43 deaths per 1000 live births, respectively, reported in the 2019 EMDHS.

Overall, 39% of the adult population has had some formal schooling. Nearly two-thirds of women (65%) had no formal schooling. Of those who have advanced to more than secondary education, less than one-third are females.

Most families live in traditional houses made of wood and mud (91%), with dirt floors (81%) and corrugated iron roofing (78%). The majority of households have access to an improved drinking water source (90%), although few have access to piped water in to their plot (18%) and the majority of households access water from public taps (57%).^22^ Only 23% of households have access to an improved latrine (Table 4), with most families having access to a pit latrine with a slab (23%) or without a slab (37%).^22^

**Table 4:** Environmental characteristics at the Birhan Health and Demographic Surveillance System, 2019

Characteristics	Measure
Water, sanitation, and hygiene	
Percent households with improved latrine	23%
Percent households with any latrine	75%
Mean walking distance to drinking water source	1.2 km
Air quality	
Percent households cook in main house	23%
Percent households with use biomass fuel for cooking	95%

Over two-thirds of residents in urban areas have access to household electricity compared with less than a third of rural residents. Two-thirds of all households have at least one family member who owns a mobile phone.

Detailed analysis of the outcomes of the pregnancy cohort will be conducted to estimate the distribution and determinants of maternal and child health and survival outcomes. Using this evidence, we will develop pregnancy and neonatal risk stratification algorithms. We hope this will improve clinical diagnosis and care in this low-resource setting.

## Strengths and weaknesses

The study area encompasses different ecological conditions with a mix of highland and lowland topography, varying weather patterns and different farming practices. The environmental, demographic and economic diversity of the site is representative of the entire Amhara Region. Variables collected are similar to national surveys and other HDSS sites around the world, which will allow for data comparisons.

Quarterly data collection is more frequent than in most global HDSS sites. This reduces the loss to follow-up and recall biases, and improves the quality of data. However, frequent rounds of data collection may risk creating community fatigue. To prevent this, we are establishing a community advisory board. This board will collaborate and engage community participation to maintain a good relationship between the study team and the community.

We have developed a de novo online electronic data system that captures longitudinal data from both household and facility visits, to accommodate low internet bandwidth. The low internet bandwidth in Ethiopia and the rural study area has created occasional difficulties with data transfer; however, the study team has adapted data transfer strategies that are offline and encrypted during internet outages. The study follows an open cohort design to capture the population dynamics in a region of the world that has historically been challenging for the conduct such type of studies. As a result, the high in- and out-migration in the area, coupled with the size of the study population, pose analytical challenges to defining a steady-state population. To calculate rates, a mid-year population was used.

## Data sharing and collaboration

Through the Birhan HDSS platform, data are jointly owned by St. Paul’s Hospital Millennium Medical College (SPHMMC) in Ethiopia and Harvard-Boston Children’s Hospital (BCH) in the USA. Birhan HDSS is open to opportunities for collaborative research both nationally and internationally. Data use is governed by the Birhan Data Access Committee (DAC) and follows Birhan’s data-sharing policy. All researchers who wish to access Birhan data can complete a Birhan data request form and submit it for decision by the Birhan DAC. Datasets will only be provided with de-identified data, to maintain confidentiality of study participants. Requests for data can be sent to the principal investigators at [birhan@sphmmc.edu.et]. Birhan serves as the field site for HaSET (‘happiness’ in Amharic)—a maternal and child health research programme in Ethiopia. For further details, please visit [www.hasetmch.org].

## Funding

This work has been supported by the Bill & Melinda Gates Foundation (grant numbers INV-010382 and OPP1201842).

## References

[1] World Health Organization. Maternal Mortality Fact Sheets.:. 2019. https://www.who.int/news-room/fact-sheets/detail/maternal-mortality (25 May 2020, date last accessed).

[2] Central Statistical Agency (CSA) [Ethiopia] and ICF. Ethiopia Demographic and Health Survey 2016. Addis Ababa, Ethiopia, and Rockville, MD: CSA and ICF, 2016.

[3] World Health Organization. *Trends in Maternal Mortality 2000 to 2017: Estimates by WHO, UNICEF, UNFPA,* World *Bank Group and the United Nations Population Division*. 2019. https://www.unfpa.org/sites/default/files/pub-pdf/Maternal_mortality_report.pdf (29 May 2020, date last accessed).

[4] United Nations Children’s Fund. Levels and Trends in Child Mortality Report *2019, Estimates Developed by the UN Inter-agency Group for Child Mortality Estimation*., 2019. https://www.unicef.org/media/60561/file/UN-IGME-child-mortality-report-2019.pdf (9 June 2020, date last accessed).

[5] Lawn J , Kerber K. *Opportunities* *for* *Africa's* *Newborns: Practical Data, Policy and Programmatic Support for Newborn Care* *in Africa*. Geneva: WHO on Behalf of the Partnership for Maternal Newborn and Child Health, 2006, p. 250.

[6] *United Nations Office for the Coordination of Humanitarian Affairs (OCHA), Ethiopia: Administrative* *Map*. 2017. https://reliefweb.int/sites/reliefweb.int/files/resources/21_adm_eth_081517_a0.pdf (2 June 2020, date last accessed)

[7] Verdin, K.L., 2017, Hydrologic Derivatives for Modeling and Applications (HDMA) database: U.S. Geological Survey data release, 10.5066/F7S180ZP (9 June 2020, date last accessed).

[8] Ethiopian Meteorological Agency. *Monthly Climatic Record of Temperature and Rainfall for Debre Birhan and Shewarobit Cities.* 2017.

[9] INDEPTH Network. *Population and Health in Developing Countries*. Vol. 1. Population, Health, and Survival at INDEPTH Sites. Ottawa, ON: IRDC, 2002.

[10] Assefa N , Berhane Y, Worku A. Pregnancy rates and pregnancy loss in Eastern Ethiopia. Acta Obstet Gynecol Scand 2013;92:642–47. 23384203 10.1111/aogs.12097

[11] Boerma JT , Black RE, Sommerfelt AE, Rutstein SO, Bicego GT. Accuracy and completeness of mothers' recall of diarrhoea occurrence in pre-school children in demographic and health surveys. Int J Epidemiol 1991;20:1073–80. 1800406 10.1093/ije/20.4.1073

[12] Byass P , Hanlon PW. Daily morbidity records: recall and reliability. Int J Epidemiol 1994;23:757–63. 8002190 10.1093/ije/23.4.757

[13] Feikin DR , Audi A, Olack B et al Evaluation of the optimal recall period for disease symptoms in home-based morbidity surveillance in rural and urban Kenya. Int J Epidemiol 2010;39:450–58. 20089695 10.1093/ije/dyp374 PMC2846445

[14] Ariff S , Lee AC, Lawn J, Bhutta ZA. Global burden, epidemiologic trends, and prevention of intrapartum-related deaths in low-resource settings. Clin Perinatol 2016;43:593–608. 27524456 10.1016/j.clp.2016.05.001

[15] Harrison LH , Moursi S, Guinena AH et al Maternal reporting of acute respiratory infection in Egypt. Int J Epidemiol 1995;24:1058–63. 8557440 10.1093/ije/24.5.1058

[16] WHO. Manual for the Training of Interviewers on the Use of the 2016 WHO VA Instrument. Geneva: World Health Organization, 2017 (10 June 2020, date last accessed).

[17] University of Washington Computer Science and Engineering Department, Open Data kit (ODK). 2020.. https://www.opendatakit.org/ (August 2020, date last accessed).

[18] Central Statistical Agency [Ethiopia] and ORC Macro. Ethiopia Demographic and Health Survey 2005. Addis Ababa and Calverton, MD: Central Statistical Agency and ORC Macro, 2006.

[19] Federal Democratic Republic Population CensusCommission. Summary and StatisticalReport of the 2007 Population and HousingCensus. Addis Ababa: CensusCommission, 2008.

[20] Central Statistical Agency. Population Projections for Ethiopia *2007–2037*., 2013. https://www.statsethiopia.gov.et/wp-content/uploads/2019/05/ICPS-Population-Projection-2007-2037-produced-in-2012.pdf (28 May 2020, date last accessed).

[21] Ethiopian Public Health Institute (EPHI) [Ethiopia] and ICF. Ethiopia Mini Demographic and Health Survey 2019: Final Report. Rockville, MD: EPHI and ICF, 2021.

[22] WHO/UNICEF. Progress on Drinking Water, Sanitation and Hygiene: 2017 Update and SDG Baselines. Geneva: World Health Organization (WHO) and the United Nations Children’s Fund (UNICEF), 2017. https://www.who.int/mediacentre/news/releases/2017/launch-version-report-jmp-water-sanitation-hygiene.pdf (9 June 2020, date last accessed).

